# Potential reversal of pulmonary vasoplegia by inhaled ibuprofenate in COVID‐19 pneumonia

**DOI:** 10.1002/ctd2.31

**Published:** 2022-02-20

**Authors:** Christian Carlos Zurita‐Lizza, Pablo Alexis Doreski

**Affiliations:** ^1^ Hospital General de Agudos Dr. Cosme Argerich Intensive Care Unit Buenos Aires Argentina; ^2^ Fundación Respirar Clinical Research Unit Buenos Aires Argentina

Dear Editor,

As recently published, the reversal of SARS‐CoV2‐induced hypoxia has been demonstrated with the administration of the non‐steroidal anti‐inflammatory drug inhaled sodium ibuprofenate in hypertonic saline formulation (NaIHS) immersed within a compassionate use program of patients with moderate and severe Coronavirus disease 2019 (COVID‐19) pneumonia in Argentina.^1^ Nonetheless, the underlying mechanisms of such effect remain unknown.^1^ Therefore, we hereby attempt to postulate the potential mechanisms upon which NaIHS exerts the aforementioned beneficial effects, possibly related to indirect inhibition of nitric oxide (NO) hyperproduction and to COVID‐19 pneumonia type L.^2^ This vasoplegic form of presentation was termed type L and may present from its onset in up to 70%–80% of cases.^2^ In the above‐mentioned compassionate use program, from a total of 383 patients (15% mechanically ventilated), both ventilated and non‐ventilated groups experienced a rapid reversal of hypoxemia with normalization of respiratory rate, a decrease in hospitalization stay and a significant reduction in mortality in heterogeneous populations.^1^ These emerging data might suggest a pharmacological interaction associated with the short elimination half‐life of gaseous NO, which is 1–5 s, and its harmful effect is concentration‐dependent in micromolar range. It is noteworthy that patients with pneumonia type L might benefit from such treatment and be non‐respondent to inhaled NO (iNO).^2^ Furthermore, patients with acute respiratory distress syndrome might improve with iNO^2^ or inhaled prostacycline in the presence of pulmonary hypertension, although further studies are required.

NO hyperproduction, the main vasoplegic mediator in experimental sepsis, will be described as one of its most likely determinant factors implicated in pneumonia type L, through the probable overexpression and dysregulation of NO‐generating enzymes like inducible NO synthase (iNOS) and indoleamine‐2,3‐dioxygenase‐1 (IDO). NaIHS may inhibit NO hyperproduction mainly due to its anti‐inflammatory and immunomodulatory effects. It may avoid the hyperproduction of NO by indirectly inhibiting iNOS and IDO through different mechanisms, such as negatively modulating the inflammatory signalling pathways cyclooxygenase‐2 (COX2), nuclear factor kappa light chain enhancer of activated B cells (NF‐kB), signal transducer and activator of transcription 3 (STAT3), toll‐like receptor 4 (TLR4), kynurenine, phosphoinositide 3‐kinase/protein kinase B/mammalian target of rapamycin (PI3K/AKT/mTOR) and the pro‐inflammatory cytokines Interleukin‐1 beta/‐one beta. (IL‐1β), tumour necrosis factor alpha (TNF‐α), Interleukin‐6/‐six (IL‐6), transforming growth factor‐beta 1 (TGF‐β1) and other important effects that will be further developed. Thus, NaIHS may lead to down‐regulation of NO hyperproduction at monocyte and endothelial level.

We will hereby briefly describe the main pathological mechanisms related to NO hyperproduction and the potential therapeutic beneficial effects of NaIHS (Figures 1 and 2 and Table 1).

**FIGURE 1:**
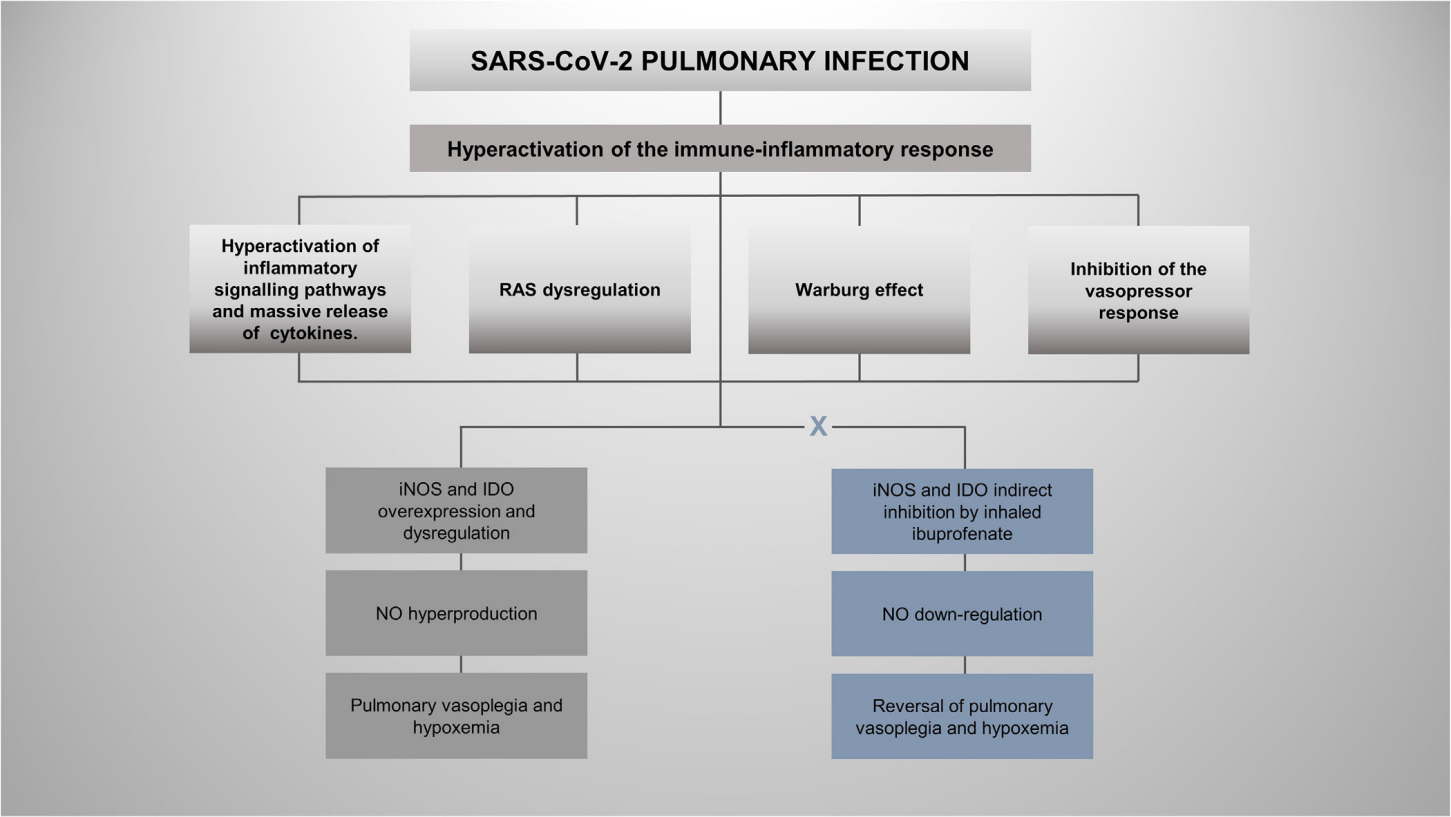
Pulmonary vasoplegia and hypoxemia mediated by nitric oxide (NO) hyperproduction could be explained by different pathological mechanisms. One arm shows preliminary evidence after the treatment with inhaled ibuprofenate (NaIHS), and the other arm shows evidence without the adjuvant treatment

**FIGURE 2:**
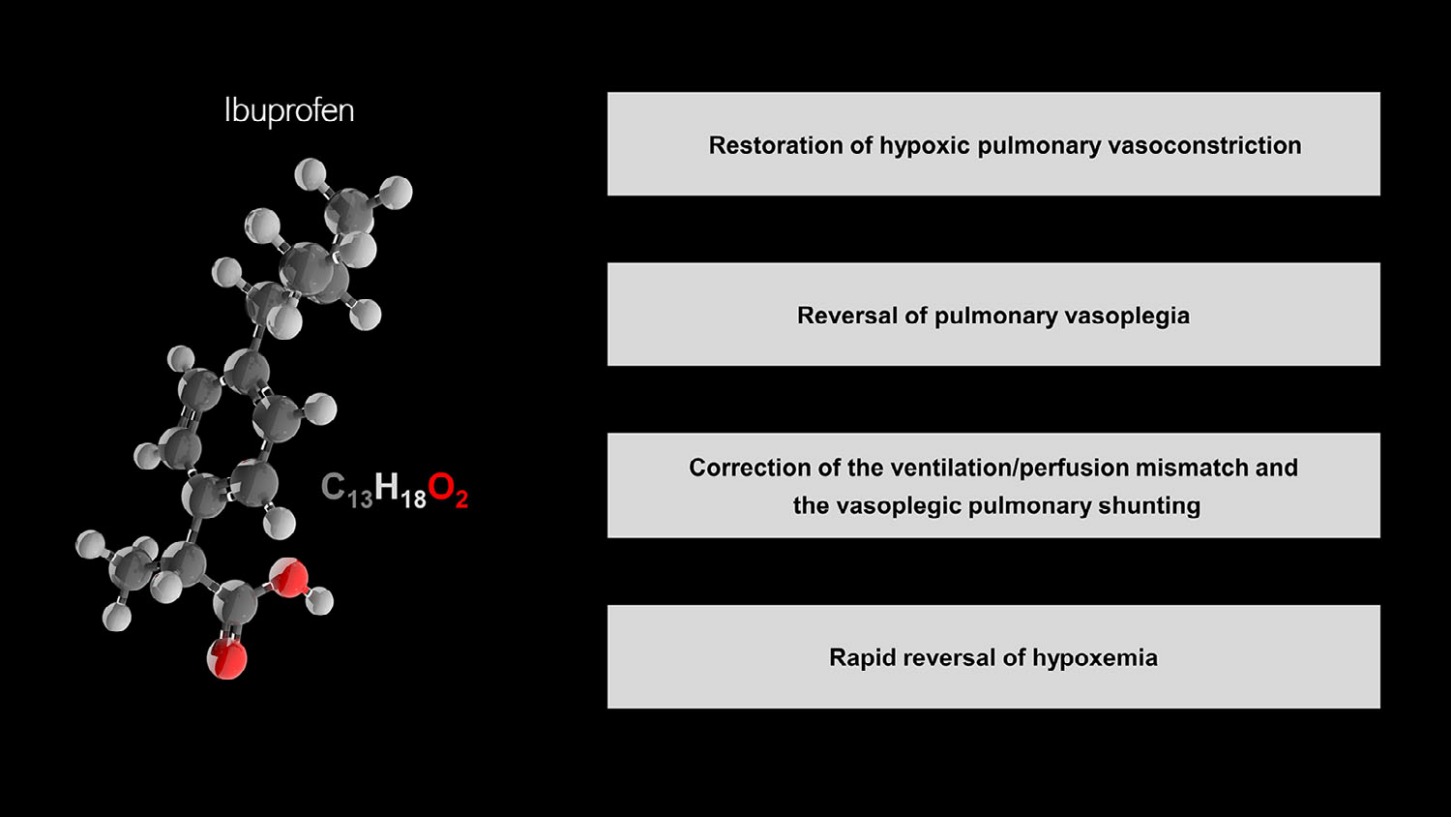
Potential anti‐inflammatory and immunomodulatory effects of inhaled Ibuprofen in hydrosoluble formulation in COVID‐19 pneumonia type L

**TABLE 1:** Potential therapeutic effects of inhaled ibuprofenate leading to down‐regulation of nitric oxide (NO) hyperproduction through the indirect inhibition of inducible NO synthase (iNOS) and indoleamine‐2,3‐dioxygenase‐1 (IDO)

Therapeutic targets	Mechanisms of action	Mechanisms of action
RAS dysregulation and iNOS^a^	Inhibition of COX‐dependent effect	Inhibition of NF‐kB, COX2 and TGF β1
Positive feed‐back loop between inflammatory signalling pathways and pro‐inflammatory cytokines and iNOS^b^	Inhibition of COX‐dependent effect	Inhibition of NF‐kB, STAT3 and IL‐6
Warburg effect and iNOS^c^	Inhibition of COX‐dependent effect	Inhibition of PI3K/AKT/mTOR
Blockade of calcium entry into vascular smooth muscle cells and iNOS^d^	Inhibition of COX‐dependent effect	Inhibition of NF‐kB, COX2, TNF‐α, IL‐1β and IL‐6
IDO^e^	Inhibition of COX‐dependent effect	Inhibition of STAT3, COX 2, NF‐kB, TLR4, IL1‐β TNF‐α and IL‐6

Abbreviations: COX2, cyclooxygenase‐2; mTOR, mammalian target of rapamycin; NF‐kB, nuclear factor kappa light chain enhancer of activated B cells; RAS, renin‐angiotensin system; STAT3, signal transducer and activator of transcription 3; TGF‐β1, transforming growth factor‐beta 1; TLR4, toll‐like receptor 4.

^a^ Restoration of RAS dysregulation^5^ by inhibition of NF‐kB,^5^ COX2^5^ and TGF‐β1^5^

^b^ Avoidance of the positive feed‐back loop between STAT3,^5^ NF‐kB^5^ and mainly IL‐6^5^

^c^ Inhibition of the Warburg effect via COX‐dependent effect PI3K/AKT/mTOR^9^

^d^ Restoration of vasopressor effect by inhibition of NF‐kB,^5^ COX2,^5^ TNF‐α,^5^ IL‐1β^5^ and IL‐6^5^ and

^e^ Indirect inhibition of IDO^4^ by negative modulation of STAT3,^5^ COX2,^5^ NF‐kB,^5^ TLR4,^5^ IL‐1β,^5^ TNF‐α^5^ and IL‐6.^5^

The generalized vascular hyperinflammatory status may lead to endothelial overexpression of IDO and STAT3 and may activate the IDO and the IDO‐kynurenine pathway.^3^ Under anaerobic conditions, the IDO‐kynurenine pathway may produce the degradation of L‐tryptophan amino acid, leading to the production of its catabolite, kynurenine, promoting up‐regulation of NO.^3^ The activation of IDO may be due to positive modulation of the COX2,^4^ NF‐kB,^3^ STAT3,^3^ TLR4^3^ and the cytokines IL‐1β,^4^ TNF‐α^4^ and IL‐6.^3^ NaIHS may produce the indirect inhibition of IDO^4^ through negative modulation of the COX2,^5^ NF‐kB,^5^ STAT3,^5^ TLR4^5^ and the cytokines IL‐1β,^5^ TNF‐α^5^ and IL‐6.^5^ Likewise, by negatively modulating the IDO‐kynurenine pathway.

Renin‐angiotensin system (RAS) dysregulation is a key factor in the production of endothelitis and generalized endothelial dysfunction. It may produce angiotensin converting enzyme 2 (ACE2) down‐regulation and angiotensin 2 (ang II) up‐regulation.^6^ ACE2 down‐regulation may promote the increase of iNOS expression with up‐regulation of NO via L‐arginine as its substrate, the decrease of endothelial NO synthase expression, the increase of reactivity of vascular smooth muscle to NO and the increase of the cytokines IL‐6 and TNF‐α.^6^ Ang II up‐regulation may produce NF‐kB activation through angiotensin receptor type 1, promoting iNOS and COX2 induction and expression, the increase of cytokines IL‐1β, TNF‐α, IL‐6 and others.^5^ NaIHS may restore RAS dysregulation,^5^ possibly by direct inhibition of NF‐kB,^5^ COX2^5^ and TGF‐β1,^5^ and thus, it may indirectly inhibit iNOS induction.

The massive cytokine release by monocytes may be amplified by a positive feed‐back loop among STAT3, NF‐kB and mainly the cytokine IL‐6, also thereby amplifying the immune‐inflammatory response and also possibly inducing iNOS.^7^ NaIHS may avoid the positive feed‐back loop between STAT3,^5^ NF‐kB^5^ and mainly the cytokine 6.^5^ In this way, NaIHS may indirectly inhibit iNOS induction.

The Warburg effect in COVID‐19 sepsis is activated in the presence of hypoxemia and thus, generates mitochondrial dysfunction and promotes the switch to oxidative metabolism, blocking the Krebs cycle, intended to obtain energy, and opting the inflamed cells for an alternative pathway to obtain quicker energy: altered aerobic glycolysis with lactate production, fatty‐acid synthesis and glutaminolysis.^8^ Moreover, the Warburg effect may possibly be activated, by positive modulation, by M1 macrophages through the PI3K/AKT/mTOR pathway, inducing iNOS.^8^ NaIHS may inhibit the Warburg effect, negatively modulating the PI3K/AKT/mTOR pathway,^9^ and thus, it may indirectly inhibit iNOS induction.

Possible inhibition of the vasopressor response by avoiding the coupling necessary for calcium entry into vascular smooth muscle cells, mainly by iNOS induction through NF‐kB, COX2 and cytokines TNF‐α, IL‐1β, IL‐6 and others.^10^ NaIHS may restore the vasopressor effect through the reversal of the blockade of calcium entry into vascular smooth muscle cells. By negative modulation of NF‐kB^5^ and COX2^5^ and the cytokines TNF‐α,^5^ IL‐1β^5^ and IL‐6,^5^ it may indirectly inhibit iNOS induction.

We have herein postulated the potential mechanisms upon which NaIHS might promote reversal of hypoxemia in COVID‐19 pneumonia type L that seem to be mainly related to indirect inhibition of NO hyperproduction. In this sense, NaIHS might promote restoration of hypoxic vasoconstriction, reversal of vasoplegia, correction of the decreased ventilation/perfusion mismatch and the vasoplegic shunting and therefore, the reversal of hypoxemia. On the basis of the above mentioned effects and of future randomized clinical trials, NaIHS might emerge as a potential adjuvant for COVID‐19 pneumonia.

## CONFLICT OF INTEREST

The authors declare that they have no conflict of interest to this work.

## FUNDING INFORMATION

None.
